# Effects of Aqueous Extract of *Schizandra Chinensis Fruit* on Cadmium-Induced Change of Monoamine Neurotransmitters in Rats

**DOI:** 10.5487/TR.2009.25.1.017

**Published:** 2009-03-01

**Authors:** Zheng Lin Zhao, Guang Wen Zhao, Li Li, Meng Quan Li, Li Xin Guan, Xu Dong Yang, Hou Zhong Li, Feng Lin, Jong Rok Lee, Rong Jie Zhao

**Affiliations:** 13 Department of Biochemistry, Mudanjiang Medical University, Mudanjiang, 157011 P.R. China; 23 Department of General Surgery, Yanbian Medical College, Yanbian University, Yanji, 133000 China; 33 Department of Pharmacology, Mudanjiang Medical University, 3 Tongxiang Street, Mudanjiang, 157011 China; 43 The Research Center for Biomedical Resource of Oriental Medicine, Daegu Haany University, Daegu, 706-828 Korea

**Keywords:** *Schizandra Chinensis Fruit*, Cadmium, Monoamines, Brain, Rat

## Abstract

The effects of aqueous extract of *Schizandra Chinensis Fruit* (AESC) on cadmium-induced changes of monoamine neurotransmitters in the different brain regions of adult rats were investigated. Male rats were received intraperitoneal (i.p.) administration of CdCl_2_ (0.6 mg/kg/d) for 21 days and sacrificed 7 days after the last administration. Concentrations of norepinephrine (NE), dopamine (DA) in striatum and serotonin (5-HT), 5-hydroxyindole acetic acid (5-HIAA) in cortex were measured by HPLC. There were significant decreases of NE, DA, 5-HT and 5-HIAA in Cd intoxicated rats (*P* < 0.05), while pretreatment with AESC (20 mg/kg/d or 60 mg/kg/d, p.o., 30 min before CdCl_2_) greatly inhibited the decrease of monoamine transmitters, respectively (*P* < 0.05). Also, AESC significantly increased the reduction of glutathione contents and superoxide dismutase activities in cortex induced by CdCl_2_. These results suggest that AESC ameliorates Cd-induced depletion of monoamine neu-rotransmitters in brain through its antioxidant activity.

## INTRODUCTION

Cadmium, a common environmental heavy metal pollutant, is noxious to many organic tissues, including kidney, lung and brain tissues (Nawrot *et al.*, 2006; Rigon *et al.*, 2008). In the central nervous system (CNS), Chronic Cd can cause neurochemical and morphological changes in brain to result in lower attention, hypernociception, olfactory dysfunction and memory deficits (De Castro *et al.*, 1996). Primarily heavy metals do damage to the CNS by their ability to destroy the normal state of neurotransmitter systems in brain. In a study done by Xu *et al.*, administration of Cd can lead to decrease of norepinephrine (NE), dopamine (DA) and serotonin (5-HT) in mice brain (Xu *et al.*, 2006a).

Numerous studies show that an important mechanism of heavy metals poisoning is the imbalance between pro-oxidant and antioxidant homeostasis caused by intake of heavy metals (Stohs *et al.*, 1995; Flora *et al.*, 2008). It is well known that Cd decreases contents of antioxidant thiols and reduces activities of antioxidant enzymes in brain (Kumar *et al.*, 1996). And due to the abundance of polyunsaturated fatty acids and high rate of oxygen utilization, the tissues in brain are highly vulnerable to Cd.

The *Schizandra chinensis fruit*, one of the widely used Oriental herbs, has been used to treat various human diseases including insomnia and amnesia (Zhu, 1998). It has been reported the therapeutic role of *Schizandra chinensis fruit* in central nervous disorders is closely associated with its effect on neurotransmitter systems. Gomisin A, an important component of *Schizandra chinensis fruit* greatly improved scopolamine-induced cognitive impairments through inhibiting acetylcholinesterase activity (Kim *et al.*, 2006).

It has already been documented the antioxidant activity of *Schizandra chinensis fruit* plays a leading role in its therapeutic actions (Wang *et al.*, 1994). Schizandrin, another important component of *Schizandra chinensis fruit* greatly prevented the cold stress-induced increase of malonic dialdehyde in rat liver homogenate and inhibited non-enzymatic ascorbate-dependent lipid peroxidation in liver homogenate *in vitro* (Lupandin *et al.*, 1986).

This study was designed to investigate the effects of aqueous extract of *Schizandra chinensis fruit* (AESC) on cadmium-induced changes of monoamine neurotransmitters in the different brain regions and the possible mechanism involved in it.

## MATERIALS AND METHODS

***Preparation of AESC.*** The dried *Schizandra chinensis fruit* was purchased from a local market and ground to fine powder, and consecutively extracted under reflux with water for 1 h. The obtained water extract was evaporated under reduced pressure at temperature of 37°C and lyophilized.

***Reagents.*** Cadmium chloride, Sodium octanesulfonic acid, acetonitrile, tetrahydrofurane, NE, DA, 5-HT, and **5**-hydroxyindole acetic acid (5-HIAA) were purchased from Sigma Co. (St. Louis, MO, USA). All other drugs were of analytical or HPLC grade.

***Animals and experimental design.*** Adult male Sprague-Dawley rats (220~250 g) were obtained from the Laboratory Animal Center in Yanbian Medical College of Yanbian University (Yanji, China). The rats were individually housed in a controlled environment during all experimental treatments. Food and water were provided *ad libitum* and the rats were maintained on a 12-hour light/dark cycle. All animal procedures were approved by the Institutional Animal Care and Use Committee and were accomplished in accordance with the provisions of the NIH "Guide for the Care and Use of Laboratory Animals."

The rats were divided into four groups. Group 1: D.W. (distilled water) + saline (W + S), Group 2: D.W. + $CdCl_2$ (W + Cd), Group 3: AESC (20 mg/kg/d) + $CdCl_2$ (AESC20 + Cd), Group 4: AESC (60 mg/kg/d) + $CdCl_2$ (AESC60 + Cd). The rats were given oral administration (p.o.) of D.W. or AESC (20 mg/kg/d or 60 mg/kg/d, dissolved in D.W.), 30 min after AESC the rats were also intraperitoneally (i.p.) received saline or $CdCl_2$ (0.6 mg/kg/d, dissolved in saline) for 3 weeks. One week after the last treatment, the rats were decapitated, cortex and striatum were dissected for the preparation of tissue homogenates.

***Monoamines analysis.*** Brain samples were sonicated in 1 ml of 0.1 M $HClO_4$ for 30 s, and centrifuged for 15 min at 26,000 *g*, 4°C. Then, a 20 μl supernatant aliquot was injected directly into the HPLC with a coulmoetric detector (Coulochem II; ESA, Bedford, MA, USA). The HPLC system consisted of a C18 reverse-phase column (5 μ ODS; Altex, Ann Arbor, MI, USA) and an electrochemical transducer with a glassy carbon electrode set at 350 mV. The mobile phase was 0.163 M citric acid, pH 3.0, containing 0.02 mM EDTA with 0.69 mM sodium octanesulfonic acid as an ion-pairing reagent, 4% (v/v) acetonitrile and 1.7% (v/v) tetrahydrofurane. Peaks and values of NE, DA, 5-HT and 5-HIAA in samples were identified and calculated by comparing their retention times and peak heights with those of standards. Results were reported as ng/g wet tissue. The protein concentration in brain homogenate was determined by the method of Lowry *et al.* (Lowry *et al.*, 1951).

***Determination of antioxidant activities.*** The levels of reduced glutathione (GSH) in cortex homogenate were determined by the method of Moron *et al.* (Moron *et al.*, 1979) based on the reaction with Ellman's reagent (19.8 mg DTNB in 100 ml of 0.1% sodium citrate). The activities of superoxide dismutase (SOD) were also measured spectrophotometrically in cortex homogenate by the method of Kakkar *et al.* (Kakkar *et al.*, 1984).

***Statistical analysis.*** All data were expressed as mean ± SEM, and analyzed statistically by one-way ANOVA followed by Tukey's multiple comparison tests using SPSS software (Student's version). $P < 0.05$ was considered statistically significant.

## RESULTS

***Effects of AESC on Cd induced neurotoxicity.*** Administration of $CdCl_2$ (0.6 mg/kg/d, i.p. for 21 days) to rats led to damage to central nervous system evidenced by significant decreases of NE and DA in striatum, 5-HT and 5-HIAA in cortex as compared to those of saline treated rats ($P < 0.05$ or 0.01). Pretreatment with AESC (20 mg/kg/d or 60 mg/kg/d, po, 30 min before $CdCl_2$) greatly inhibited the decreased levels of NE, DA, 5-HT and 5-HIAA induced by $CdCl_2$ in dose dependent way (Table 1) ($P < 0.05$).

***Effects of AESC on Cd produced damage to antioxidant defense system in cortex.*** The contents of GSH and activity of SOD in cortex are important bio-

**Table 1.** Effects of AESC on Cd-induced changes of concentrations of monoamines and metabolite in rat brain

| groups | NE (striatum) | DA (striatum) | 5-HT (cortex) | 5-HIAA (cortex) |
|---|---|---|---|---|
| W + S (6) | 353.2 ± 30.3** | 4333.7 ± 348.9* | 467.2 ± 44.1* | 1102.3 ± 77.4* |
| W + Cd (7) | 228.4 ± 26.8 | 2833.6 ± 211.1 | 322.2 ± 29.3 | 772.7 ± 60.2 |
| AESC20 + Cd (6) | 286.1 ± 22.1*,# | 3624.3 ± 290.2*,# | 379.2 ± 32.7*,# | 868.3 ± 138.3 |
| AESC60 + Cd (6) | 330.3 ± 23.1* | 4220.1 ± 300.3* | 439.3 ± 33.8* | 1003.6 ± 119.5* |

NE, norepinephrine; DA, dopamine; 5-HT, serotonin; 5-HIAA, 5-hydroxyindole acetic acid. Data are presented as mean ± SEM ng/g wet tissue in the striatum or cortex from rats treated daily with $CdCl_2$ (0.6 g/kg/d, i.p.) for 21 days and sacrificed 7 days after the last administration. The numbers in parentheses indicate the number of rats in each group. *: $P < 0.05$, **: $P < 0.01$ compared to W + Cd; #: $P < 0.05$, compared to AESC60 + Cd. (ANOVA, followed by the *post hoc* Tukey test).

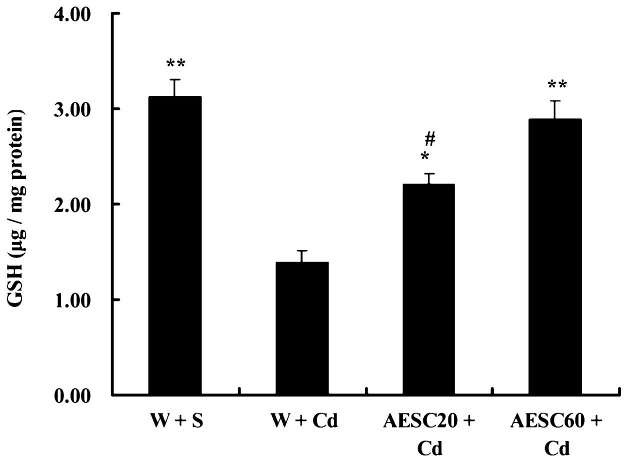

**Fig. 1.** Effects of AESC on Cd-induced reduced levels of GSH (cortex) in rats. Data are presented as mean ± SEM μg/mg protein from cortex of rats (six rats per group) 7 days after the last drugs treatment. *: $P < 0.05$, **: $P < 0.01$ compared to W + Cd; #: $P < 0.05$, compared to AESC60 + Cd. (ANOVA, followed by the *post hoc* Tukey test).

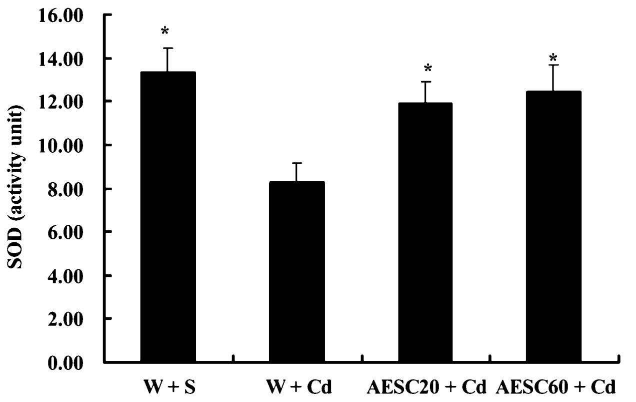

**Fig. 2.** Effects of AESC on Cd–induced reduced activities of SOD (cortex) in rats. Data are presented as mean ± SEM. The activities of SOD are expressed as follows: one unit of activity was taken as the enzyme reaction, which gave 50% inhibition of nitroblue tetrazolium reduction in 1 min/mg protein from cortex of rats (six rats per group) 7 days after the last drugs treatment. *: $P < 0.05$, compared to W + Cd (ANOVA, followed by the *post hoc* Tukey test).

chemical parameters to estimate the capacity to defense oxidative stress. Administration of $CdCl_2$ to rats resulted in significant reduction of GSH contents and SOD activities in rats as compared to those in saline treated rats ($P < 0.05$), while pretreatment with AESC reversed the alterations in dose dependent manner (Fig. 1. and Fig. 2.) ($P < 0.05$)

## DISCUSSION

Norepinephrine, dopamine and serotonin are most important classic neurotransmitters, and the alteration of their concentrations in brain is a hallmark indicating dysfunction of central nervous system. The potency of Cd as a neurotoxin has been demonstrated both in *in vitro* and *in vivo* studies (Webster and Valois, 1981; Kabeer *et al.*, 1989). Like other heavy metals, Cd disturbs metabolism of neurotransmitters and decreases NE, DA and 5-HT in various brain regions (Xu *et al.*, 2006a). In this experiment, administration of $CdCl_2$ (0.6 mg/kg/d) to rats for 21 days significantly decreased the levels of NE (in striatum), DA (in striatum), 5-HT (in cortex) and 5-HIAA (in cortex) 7 days after the last administration, these results were identical to similar studies done by other investigators (Pillai *et al.*, 2003; Xu *et al.*, 2006a).

There would be two reasons to account for reduced levels of neurotransmitters, one is down-regulation of synthesis, the other is up-regulation of elimination. 5-HIAA is a metabolized form of 5-HT, in this experiment both of 5-HT and 5-HIAA were significantly decreased in Cd treated rats as compared with saline treated rats, it indicates the decrease of neurotransmitters in brain induced by Cd very likely came from the down-regulation of synthesis. Because most of enzymes involved in synthesis of neurotransmitters in brain are vulnerable to oxidative stress, the deficiency of antioxidant molecules can lead to decrease of neurotransmitters in brain. Several studies show that the capacity of antioxidant defense system is diminished in rats exposed to Cd. Cd can penetrate the blood brain barrier, accumulate into the brain and lead to damage to antioxidant defense system in brain, as a result, there is elevation of lipid peroxidation in brain with decrease in the levels of reduced glutathione and total sulphydryl groups.

GSH is the most abundant non-protein thiol that maintains the cellular redox status and provides first line of antioxidant protection against oxidative stress in the brain (Dringen *et al.*, 2000). SOD is an important antioxidant enzyme that catalyzes the dismutation of superoxide into oxygen and hydrogen peroxide to protect cells from attack by oxidative stress. The decreased levels of GSH and reduced activities of SOD are important biomarkers to show deficiency of tissue antioxidant capacity. In this study there was significant decrease of GSH concentrations and SOD activities in rats treated with Cd. These results were also consistent with the data obtained in other studies (Murugavel and Pari, 2007).

It can be considered that Cd does damage to antioxidant defense system in brain, elevates the generation of harmful reactive oxygen species, further to disturb the activities of enzymes involved in neurotransmitters synthesis, finally cause the decrease of neurotransmitters in brain.

Recently extracts from traditional oriental herbs have increasingly been popular in treating disorders in central nervous system induced by exogenous stress or endogenous stress, and one important mechanism is associated with their ability to counter the changes of neurotransmitter systems in brain. Xu *et al.* demonstrated juice of *Hippophae Rhamnoides L* (400 g/l/d, p.o.) was effective in treating lead-induced neurotoxicity through reversing the decreased levels of NE, 5-HT and 5-HIAA in mice brain (Xu *et al.*, 2006b), and Lu *et al.* showed that Chinese mushroom (*Huangmo*) polysaccharides significantly increased the content of monoamine neurotransmitters in cerebral tissues of aging mice induced by D-galactose (Lu *et al.*, 2007). In this study prophylactic administration of AESC (20 mg/kg/d, 60 mg/kg/d, p.o.) before $CdCl_2$ dose-dependently inhibited the decrease of NE, DA, 5-HT and 5-HIAA in brain tissues.

The beneficial effects of extracts from Oriental herbs which are effective in treating heavy metal neurotoxicities are attributed to improved antioxidant activity, which potentially reduces generation of the active free radicals harmful to all cells and proteins in brain (Numagami *et al.*, 1996; Rahman, 2003). Accordingly, daily intake of tetrasulfide from garlic restored the depletion of antioxidant members in rats brain induced by Cd (Murugavel and Pari, 2007), and juice of *Hippophae Rhammoindes L* antagonized the increase of malonic dialdehyde in mice brain induced by lead (Xu *et al.*, 2006b).

Numerous studies have shown that *Schizandra chinensis fruit* is full of components with antioxidant activities. Ko *et al.* demonstrated that extract of *Schizandra chinensis fruit* decreased the elevation of malonic dialdehyde and enhanced GSH status in liver induced by $CCl_4$ (Ko *et al.*, 1995). Also dibenzocyclooctenes, lignans from *Schizandra chinensis fruit* exhibited a protective action against oxidative stress-associated ageing-related brain ischemia (Xue *et al.*, 1992). In this study AESC (20 mg/kg/d and 60 mg/kg/d) significantly ameliorated the deficiency of GSH and SOD in brain induced by Cd.

In summary, this study investigated the effect of aqueous extract of *Schizandra Chinensis fruit* on Cd-induced decrease of monoamine neurotransmitters in brain tissues and its possible mechanism. AESC (20 mg/kg/d, 60 mg/kg/d) greatly increased the reduction of neurotransmitters in brain and significantly reversed the decrease of GSH contents and SOD activities induced by Cd. It indicates that preventive administration of AESC prior to Cd can protect antioxidant system in brain against the damage coming from Cd intake, further block the decrease of monoamine neurotransmitters in brain. This study may provide a clue to the role of *Schizandra chinensis fruit* in treating Cd-induced neurotoxicity. The isolation and identification of the active component (s) of AESC and the investigation of possible effects of AESC *in vivo* need further studies.

## Abbreviations

AESC: aqueous extract of *Schizandra Chinensis Fruit*
NE: norepinephrine
DA: dopamine
5-HT: serotonin
5-HIAA: 5-hydroxyindole acetic acid
GSH: glutathione
SOD: superoxide dismutase
